# Small‐cell lung cancer from the peripheral lung is frequently accompanied by emphysema and interstitial lung disease in the background

**DOI:** 10.1111/1759-7714.14598

**Published:** 2022-07-29

**Authors:** Yuki Ikematsu, Miiru Izumi, Koichi Takayama, Hiroyuki Kumazoe, Kentaro Wakamatsu, Masayuki Kawasaki

**Affiliations:** ^1^ Department of Respiratory Medicine National Hospital Organization Omuta National Hospital Fukuoka Japan; ^2^ Department of Pulmonary Medicine, Graduate School of Medical Science Kyoto Prefectural University of Medicine Kyoto Japan; ^3^ Department of Radiology National Hospital Organization Omuta National Hospital Fukuoka Japan

**Keywords:** central type, emphysema, interstitial lung disease (ILD), peripheral type, small‐cell lung cancer (SCLC)

## Abstract

**Background:**

It has long been thought that small‐cell lung cancer (SCLC) is a central type of tumor that is located in the proximal bronchi and the mediastinum. However, several studies reported that SCLC exhibited several types of spread pattern on computed tomography (CT). The aim of this study is to investigate the relationship between CT images and clinical characteristics in patients with SCLC.

**Methods:**

We retrospectively reviewed the CT images of 92 SCLC patients and classified them into six types of spreading patterns: central, peripheral, lymphangitic spread (LYM), pleural dissemination (PLE), lobar replacement (LOB), and air‐space consolidation (AC). We also evaluated the correlation between primary tumor location and the clinical characteristics of patients.

**Results:**

The most common type of imaging pattern was peripheral (*n* = 40, 44%), with the next most common type being central (n = 27, 29%). Atypical types of SCLC, such as LYM (n = 2, 2%), PLE (n = 4, 4%), LOB (n = 8, 9%), and AC (n = 11, 12%), were also recognized in our study. The prevalence of emphysema and interstitial lung disease (ILD) was significantly higher in the peripheral type than in the central type (*p* = 0.0056 and *p* = 0.0403, respectively). Meanwhile, no survival difference was seen between the central type and the peripheral type (median months 17.9 vs. 21.9, respectively, *p* = 0.720).

**Conclusions:**

The peripheral type of tumor was correlated with higher prevalence of emphysema and ILD in SCLC. Our result suggests different mechanisms of development and tumor characteristics according to tumor location.

## Graphical abstract

In the present study, we classified computed tomography images of 92 small‐cell lung cancer patients into six types of spreading patterns: central, peripheral, lymphangitic spread, pleural dissemination, lobar replacement, and air‐space consolidation. The most common type of imaging pattern was peripheral, and the prevalence of emphysema and interstitial lung disease was significantly higher in the peripheral type than in the central type.

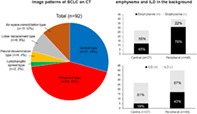

## INTRODUCTION

Small‐cell lung cancer (SCLC) is a high‐grade neuroendocrine (NE) lung carcinoma that accounts for 10%–15% of all lung cancers.^1^ It was originally classified as mediastinal carcinoma, which arose from the bronchial origin of oat cells or Kultschitzky cells that presented with NE properties and contained neuronal enzymes.^2^ It has therefore long been thought that SCLC is a central type of tumor that originates from proximal bronchi or the adjacent mediastinum.^3^ Currently, further studies in mouse models and cell lines have provided evidence that pulmonary neuroendocrine cells that arose from local multipotent stem cells are the likely origin of SCLC.^4^ A few recent studies have shown that the primary tumor origin of SCLC was predominantly the peripheral lung.^5^, ^6^ These studies have also suggested that the tumor location of origin may be of prognostic relevance in SCLC patients, although a conclusion has not yet been reached.

SCLC is also known to be strongly associated with smoking. Additionally, cigarette smoking is an important contributor to chronic obstructive pulmonary disease as well as interstitial lung disease (ILD), such as idiopathic pulmonary fibrosis (IPF), which plays an important role in increasing the incidence of lung cancer.^7^, ^8^ Thus, emphysema and ILD can be frequently detected in patients with SCLC, but little is known about the association between these clinical findings and the imaging appearance of SCLC on computed tomography (CT).

In the present study, we investigated the correlation between the imaging patterns of SCLC patients and clinical findings, such as coexisting emphysema and ILD, and prognosis.

## METHODS

### Patients

Ninety‐two patients who were diagnosed with pure SCLC in the National Hospital Organization Omuta National Hospital from June 2009 to September 2020 were included. In our study, patients who were diagnosed with combined SCLC such as combined small‐cell and non‐small‐cell lung cancer were excluded. The diagnoses of 92 patients were based on the WHO classification of lung tumors.^9^ Characteristics of the patients including age, sex, smoking history, presence of emphysema or ILD, disease stage, and survival time were extracted from the medical records. The purpose of our study was to examine the correlation between the imaging patterns of SCLC patients and clinical characteristics, including prognosis. For avoiding the bias for therapeutic agents, we excluded patients who received antibodies for programmed cell death‐ligand 1 (PD‐L1), which have been approved with chemotherapy since 2017. The present study was approved by the Ethics Committee of the National Hospital Organization of Omuta Hospital.

### Definition of image patterns of SCLC, emphysema, and ILD

**FIGURE 1:**
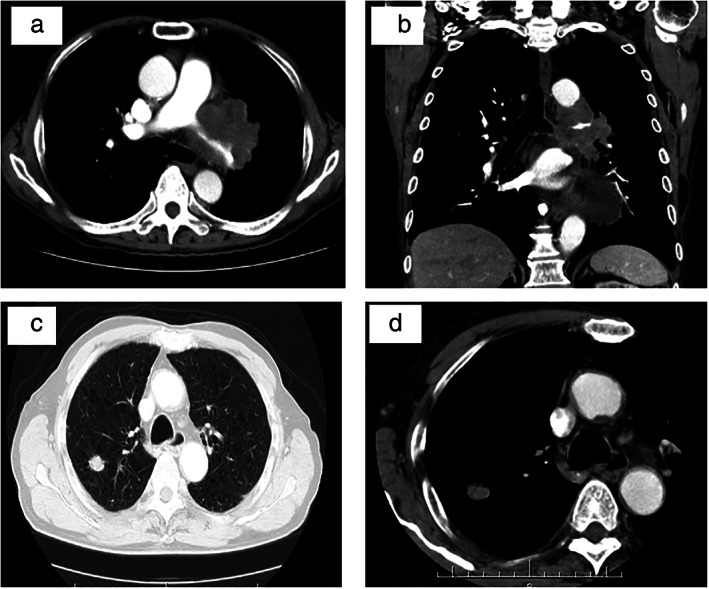
Representative images of the tumor extension and spread pattern of central and peripheral types of small‐cell lung cancer. Typical thin‐sliced computed tomography images of the central (a and b) and peripheral (c and d) types

Based on previous studies,^3^, ^10^ we retrospectively reviewed the CT images of 92 SCLC patients at diagnosis and classified them according to the primary tumor location and spreading pattern. Primary tumors involving segmental or more proximal bronchi, including ipsilateral mediastinum, were defined as the central type (Figure 1a,b). On the other hand, primary tumors involving segmental or more distal bronchi were defined as the peripheral type (Figure 1c,d). Peripheral tumors with mediastinum lymphadenopathy were also included in peripheral type. Tumors with an unclear primary location were defined as atypical types, such as the lymphangitic spread (LYM), pleural dissemination (PLE), lobar replacement (LOB), and air‐space consolidation (AC).

**FIGURE 2:**
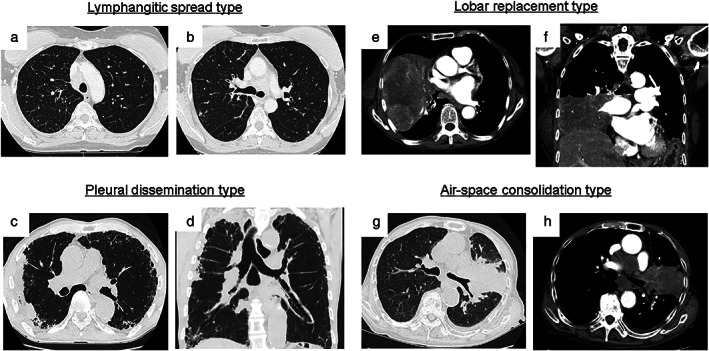
Representative images of the tumor extension and spread pattern of atypical types of small‐cell lung cancer. Typical thin‐sliced computed tomography images of the lymphangitic spread type (a and b), pleural dissemination type (c and d), lobar replacement type (e and f), and air‐space consolidation type (g and h)

Tumors with thickening of broncho‐vascular bundles and interlobular septal lines were defined as the LYM type (Figure 2a,b). Multiple subpleural small tumors associated with malignant pleural effusion were defined as the PLE type (Figure 2c,d). Huge tumors entirely replacing one lobe were defined as the LOB type (Figure 2e,f). Tumors of poorly marginated consolidation with air‐bronchogram on CT were defined as the AC type (Figure 2g,h).

Emphysema was characterized on CT images by low‐attenuation regions that contrast with surrounding normal parenchyma. ILDs were reviewed based on the CT findings at diagnosis of SCLC in accordance with the official guidelines of the American Thoracic Society and European Respiratory Society.^11^ The retrospective radiographic review and classification of image patterns of SCLC, emphysema, and ILD on CT were performed independently by two pulmonologists (Y.I. and M.I.) and one radiologist (H.K.).

### Statistical analysis

Correlation of patient characteristics to the central or peripheral type of SCLC was evaluated with the chi‐square test. Overall survival (OS) was measured from the date of SCLC diagnosis to death or the last follow‐up. OS was evaluated with the Kaplan–Meier method, and the log‐rank test was applied to compare the cumulative survival times between patient groups. A *p* value of <0.05 was considered statistically significant. All statistical analyses were performed with GraphPad Prism 7 software (GraphPad Software).

## RESULTS

### Clinical characteristics of patients and tumors

Between June 2009 and September 2020, we identified 92 patients who were diagnosed with SCLC. The patients' clinical characteristics are summarized in Table 1.

**TABLE 1:** Characteristics of 92 study patients (median age 72 years, with a range of 55–94 years)

Characteristic	*n*	(%)
Sex		
Male	72	(78.3)
Female	20	(21.7)
Smoking history		
Smoker	85	(92.4)
Never‐smoker	7	(7.6)
Stage		
I	12	(13.0)
II	9	(9.8)
III	34	(37.0)
IV	37	(40.2)
Emphysema		
Yes	57	(62.0)
No	35	(38.0)
ILD		
All	25	(27.2)
UIP	17	(18.5)
Non‐UIP	8	(8.7)
None	67	(72.8)
Treatment		
Surgery	11	(12.0)
Radiotherapy	1	(1.0)
Chemoradiotherapy	11	(12.0)
Chemotherapy	55	(59.0)
None	15	(16.0)

*Abbreviations*: ILD, interstitial lung disease; UIP, usual interstitial pneumonia.

**FIGURE 3:**
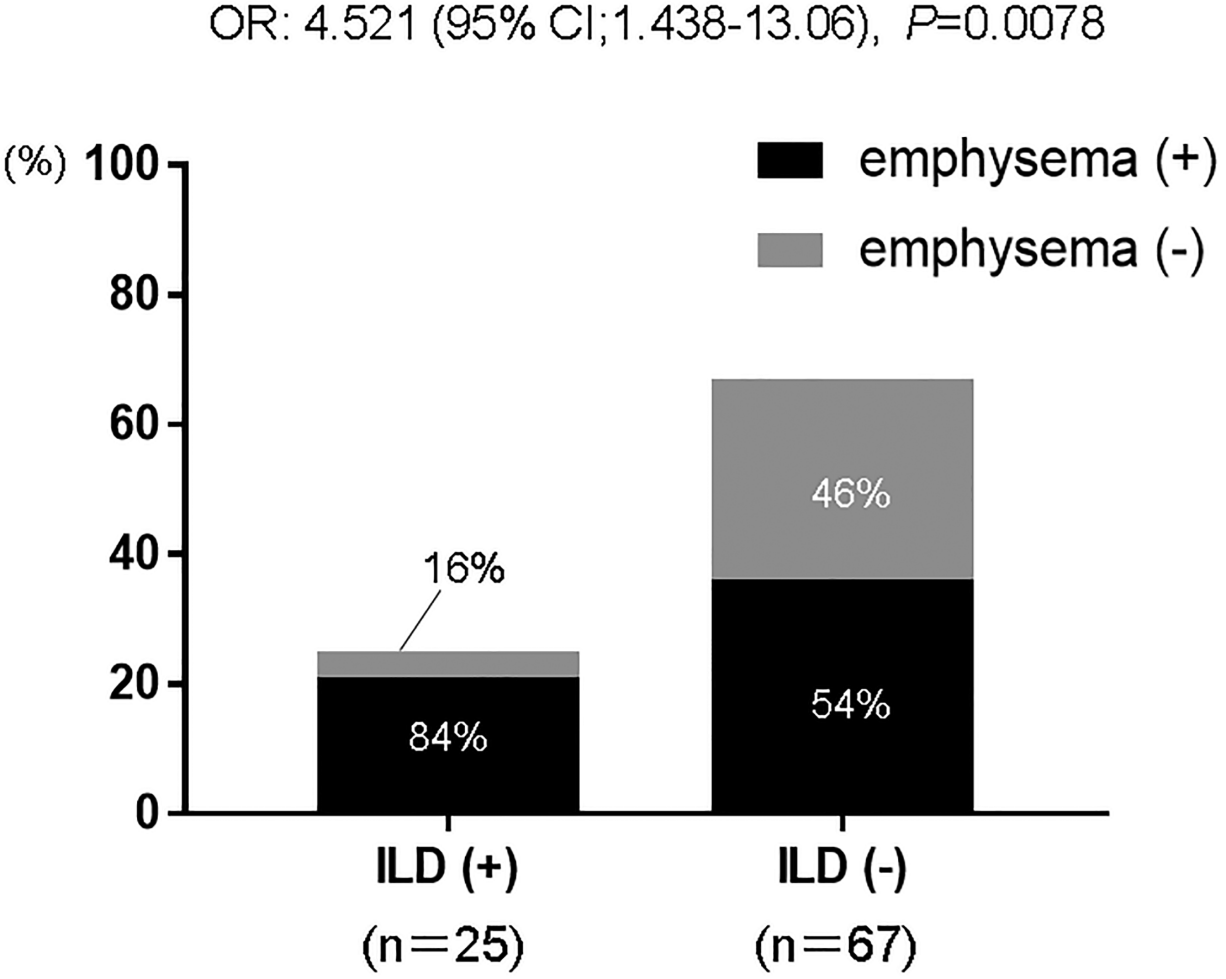
Correlation between emphysema and interstitial lung disease (ILD) in patients with small‐cell lung cancer (SCLC). Emphysematous changes were seen more often in SCLC patients with ILD than in non‐ILD patients (odds ratio [OR] 4.521, *p* = 0.0078). CI, confidence interval

The median age of all patients was 72 years (range 55–94 years). Seventy‐two patients (78.3%) were male and 85 (92.4%) were current or former smokers. Regarding the clinical stages of SCLC, 71 patients (77.2%) were stage III or IV. Fifty‐seven patients (62.0%) were diagnosed with emphysema. Among 25 patients (27.2%) with ILD, 17 were recognized as having ILD with the usual interstitial pneumonia (UIP) pattern and the remaining eight patients were recognized as having a non‐UIP pattern. In our study, emphysematous changes were seen more often in SCLC patients with ILD than in non‐ILD patients (odds ratio of 4.521, *p* = 0.0078) (Figure 3). In regard to treatment, 11 patients (12%) received surgery, one patient (1%) received radiotherapy, 11 patients (12%) received chemoradiotherapy, and 55 patients received chemotherapy (59%).

### Image patterns of SCLC on CT and the relationship between tumor location and clinical characteristics

**FIGURE 4:**
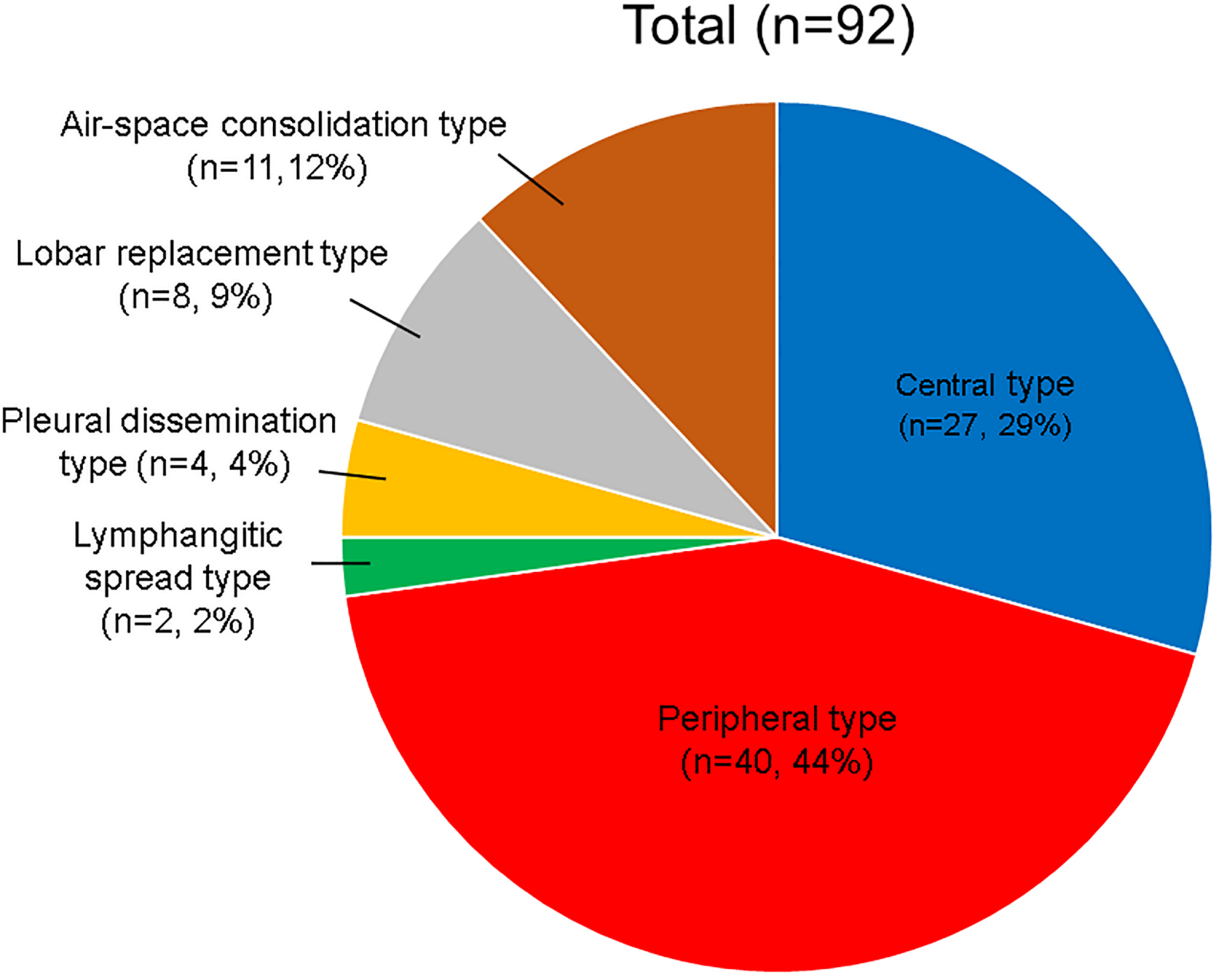
Ninety‐two small‐cell lung cancer patients classified into six categories based on image findings

Among the 92 SCLC patients classified into six different types of CT images, 27 were the central type (29%), 40 were the peripheral type (44%), two were the LYM type (2%), four were the PLE type (4%), eight were the LOB type (9%), and 11 were the AC type (12%) (Figure 4). As shown in Figure 3, the largest number of patients had the peripheral type. We next evaluated the relationship of patient characteristics to the main two types—central or peripheral—in SCLC (Table 2). No significant association was apparent between the central or peripheral type and either age, sex, smoking history, or disease stage. The mean value of pro‐gastrin‐releasing peptide (Pro‐GRP), which is a specific tumor marker for SCLC, also did not differ between the central and peripheral types (331.1 pg/mL vs. 249.9 pg/mL, respectively, *p* = 0.727). On the other hand, the prevalence of emphysema and ILD was significantly higher in the peripheral group than in the central group (emphysema: odds ratio of 4.306, *p* = 0.0056; ILD: odds ratio of 3.252, *p* = 0.0403).

**TABLE 2:** Correlation between central or peripheral type of SCLC and clinical characteristics of patients

	Tumor location (*n*, %)				
Characteristic	Central type (*n* = 27)	Peripheral type (*n* = 40)	Total (*n* = 67)	OR (95% CI)	*p* value
Age (years)					
<75	13	25	38	1.795 (0.652–4.531)	0.2448
≥75	14	15	29		
Sex					
Male	20	34	54	1.983 (0.595–6.68)	0.267
Female	7	6	13		
Smoking history					
Former	26	39	65	1.5 (0.0763–29.14)	0.776
Never	1	1	2		
Stage					
LD	10	18	28	1.391 (0.504–3.619)	0.5169
ED	17	22	39		
Emphysema					
Yes	12	31	43	4.306 (1.42–12.1)	0.0056
No	15	9	24		
ILD					
Yes	5	17	22	3.252 (1.084–9.123)	0.0403
No	22	23	45		

*Abbreviations*: CI, confidence interval; ED, extensive disease; ILD, interstitial lung disease; LD, limited disease; OR, odds ratio; SCLC, small‐cell lung cancer.

### Comparison of OS according to tumor location as well as treatment management in SCLC

**FIGURE 5:**
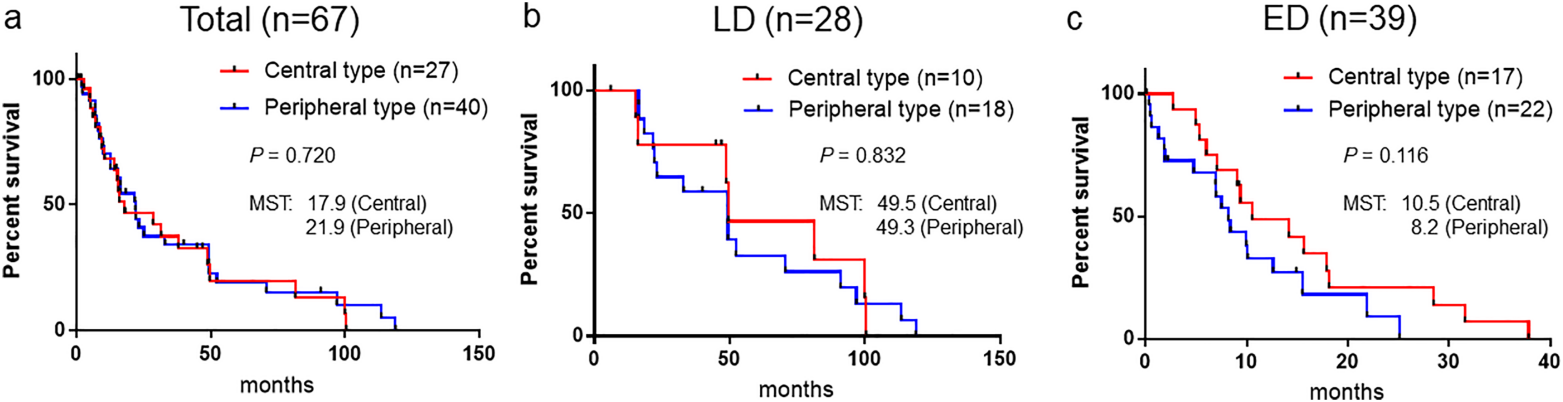
Kaplan–Meier survival curves comparing the central and peripheral types in small‐cell lung cancer patients. (a) All cases (*n* = 67, log‐rank test, *p* = 0.720), (b) limited disease (LD) cases (*n* = 28, log‐rank test, *p* = 0.832), and (c) extensive disease (ED) cases (*n* = 39, log rank test, *p* = 0.116). MST, median survival time

Because few studies have reported survival differences in patients with SCLC according to tumor location, we analyzed these comparison of OS as well as treatment management between the central and peripheral types. Compared to the central type, the rate of surgery was higher in the peripheral type of SCLC (3.7% vs. 22.5%, respectively) (Table 3). On the other hand, patients who received chemotherapy were more seen in central type than in peripheral type (70.4% vs. 50.0%, respectively). The median survival time (MST) was 17.9 months for the central type and 21.9 months for the peripheral type, but no significant difference in OS was apparent between the two types (*p* = 0.720) (Figure 5a). We then divided SCLC patients into limited disease (LD) and extended disease (ED) groups to determine whether there was a difference in the prognoses between the central and peripheral types (Figure 5b,c). Although no significant difference was observed, the peripheral type of ED‐SCLC tended to have poorer prognoses compared to the central type (MST 8.2 vs. 10.5 months, respectively, *p* = 0.116) (Figure 5c). We further evaluated the presence of emphysema, ILD, the number of treatment lines, and the rate of acute exacerbation of ILD or drug‐induced pneumonia in ED‐SCLC patients (Table 3). The prevalence of emphysema was significantly higher in the peripheral type of ED‐SCLC patients (odds ratio 18.67, *p* = 0.002). Although the differences were not statistically significant, an increased presence of ILD, fewer treatment lines, and a higher rate of acute exacerbation of ILD or drug‐induced pneumonia were observed more often in the peripheral type as compared to the central type.

**TABLE 3:** Differences in clinical characteristics and management between central‐ and peripheral‐type SCLC patients

		Total, *n* = 67	Central, *n* = 27 (%)	Peripheral, *n* = 40 (%)		
First treatment						
	Surgery		1 (3.7)	9 (22.5)		
	Radiotherapy		0 (0)	1 (2.5)		
	Chemo‐radiotherapy		6 (22.2)	5 (12.5)		
	Chemotherapy		19 (70.4)	20 (50.0)		
	None		1 (3.7)	5 (12.5)		
		ED, *n* = 39	Central, *n* = 17 (%)	Peripheral, *n* = 22 (%)	OR (95% CI)	*p* value
Emphysema					18.67 (2.48–214.7)	0.002
	Yes		9 (52.9)	15 (68.2)		
	No		8 (47.1)	7 (31.8)		
ILD					2.0 (0.531–7.48)	0.307
	Yes		5 (29.4)	10 (45.6)		
	No		12 (70.6)	12 (55.4)		
Number of treatment lines					1.905 (0.154–1.986)	0.332
	0 or 1		9 (52.9)	15 (68.2)		
	>2		8 (47.1)	7 (31.8)		
						
Drug‐induced pneumonia or exacerbation of ILD					3.556 (0.472–45.9)	0.255
	Yes		1 (5.9)	4 (18.2)		
	No		16 (94.1)	18 (81.8)		

*Abbreviations*: CI, confidence interval; ED, extensive disease; ILD, interstitial lung disease; OR, odds ratio; SCLC, small‐cell lung cancer.

## DISCUSSION

Our study has shown that the most common type of SCLC imaging pattern was the peripheral type. Our results support recent studies that show that the frequency of SCLC occurring in the peripheral lung is dominant, indicating that the apparent sites of origin for SCLC might be different from classical theories suggesting that most SCLC arises in central areas of the lung or at the mediastinum.^12^, ^13^ The possible reasons for the higher frequency of peripherally located SCLC might be differences in ethnicity^14^ and the development of high‐resolution CT that enables us to observe the peripheral lung field without any slice gap. In addition, the original sites of SCLC in the peripheral lung are often smaller than metastases of mediastinal sites, thus physicians might have misunderstood mediastinal metastases as tumors of central origin.

Similar to the previous study,^10^ atypical imaging patterns of SCLC, such as LYM, PLE, LOB, and AC, were also recognized in our study. The present study demonstrates that SCLC sometimes spreads via the lymphatic system, interstitial space, and pleura, and SCLC can exhibit a variety of forms of invasion, spread, and extension.

We further evaluated the correlation between tumor location and emphysema or ILD in patients with SCLC. As a result, a higher prevalence of emphysema or ILD was associated more with the peripheral type of SCLC than with the central type. Although previous reports have showed that patients with emphysema have a higher proportion of centrally located lung cancers, such as squamous cell carcinoma, severe emphysema was correlated with a peripheral location of lung cancer.^15^ Another study also reported that squamous cell carcinoma was the most common type of lung cancer with a central location; however, these tumors in peripheral lesions were more frequently seen in emphysematous areas.^16^ In our study, patients with SCLC of peripheral origin might have severe emphysematous changes compared to those with SCLC of central origin. ILD is characterized by chronic damage to the alveolar epithelium associated with profound changes in the alveolar structure and fibrosis. Previous reviews showed that lung cancer with ILD mostly manifests as peripheral tumors (83.9%) developing within or near fibrotic areas (68.1%) in the inferior lung lobes (58.7%)^17^; this supports our result of a higher proportion of ILD in the group with peripheral SCLC than in the central group. In our study, most ILD in patients with SCLC was classified as having a UIP pattern. Furthermore, ILD patients were significantly associated with the coexistence of emphysema. Because SCLC, IPF, and emphysema patients have common risk factors, such as being male and having a history of heavy smoking, combined pulmonary fibrosis and emphysema, which is characterized by upper lobe emphysema as well as lower lobe fibrosis, may be more frequently observed in ILD patients with SCLC.^18^ Interestingly, previous paired analysis of the tumor mutation burden (TMB) for fibrosing lung tissue and tumor samples from patients with IPF‐associated adenocarcinoma demonstrated that the TMB for IPF‐associated lung adenocarcinoma was significantly higher than that for matched IPF tissue.^19^ Furthermore, somatic variants were rarely shared between the tumor and corresponding IPF tissues, and the authors concluded that not only an accumulation of somatic mutations but also various other factors, such as inflammation and oxidative stress, might be responsible for the development of adenocarcinoma with IPF. In our study, emphysema was more common in SCLC patients with ILD than in non‐ILD patients. Therefore, in the case of SCLC with ILD, the chronic inflammation of emphysema may accelerate the TMB as well as somatic mutations and promote the carcinoma. Further studies, including the genomic sequencing of SCLC with ILD, will be required to reveal the mechanism of development for such tumors.

Recent comprehensive genomic analysis of central and peripheral types of SCLC revealed that the TMB of the peripheral type was higher than that of the central type.^20^ Furthermore, somatic copy number alternation regions and recurrent amplification cytobands or genes were different between the central and peripheral types. This recent study indicates that these mutations lead to different effects in immunotherapy and tumorigenesis. In our study, peripheral SCLC was frequently accompanied by emphysema and ILD, and the chronic damage or inflammation may increase the TMB as well as somatic mutations. Although a conclusion has not yet been reached, we speculate that the mechanism of development and the characteristics of tumors may differ depending on the primary tumor location. In addition to that, understanding the difference in genomic mutations and clinical findings according to tumor location can be useful to establish therapeutic targets and additional prognostic indicators.

A few previous studies have mentioned the prognostic value from the viewpoint of tumor origin, whether in the central group or the peripheral group. One previous study reported that the peripheral type of SCLC had a worse prognosis than that the central type of tumors, and thyroid transcription factor‐1 (TTF‐1) expression was significantly correlated with the peripheral location.^5^ However, another study indicated that the peripheral type of SCLC was associated with longer survival, even though there was a higher proportion of ILD in the peripheral type of SCLC than that in the central type.^6^ Meanwhile, another study suggested that there was no survival difference between the two types.^3^ Several potential reasons for these discrepancies might be differences in sample sizes, treatments, and complications. In our study, even if SCLC patients were divided into LD or ED groups, there was no difference in prognosis due to the location of the tumor. However, the peripheral type of ED‐SCLC tended to have poorer prognoses compared to the central type. The reasons for this are presumed to be that the peripheral type is often accompanied by emphysema and ILD in the back ground, the number of treatment lines is small, and the rate of acute exacerbation of ILD or drug‐induced pneumonia is high. Previous studies reported that the presence of ILD or severe emphysema was a poor prognostic factor in SCLC patients,^6^, ^21^ and our study showed that SCLC patients with ILD were significantly associated with worse survival than those with non‐ILD (median 14.2 vs. 37.9 months, respectively, *p* = 0.004). SCLC patients with emphysema also tended to have poorer prognoses compared to those with non‐emphysema (14.2 vs. 37.9 months, respectively, *p* = 0.085). Furthermore, some of these peripheral‐type patients could not receive key chemotherapeutic agents such as irinotecan and amrubicin because of the contraindication to IPF patients. Further prospective studies using large numbers of SCLC cases, including those receiving immunotherapy, may lead to the discovery of additional prognostic factors from the viewpoint of tumor location.

With regard to limitations, this study was conducted based on a retrospective review of medical records, and a potential selection bias exists because our study was conducted at a single institution. In addition, emphysema and ILD were diagnosed based only on radiological imaging on CT.

As far as we know, this is the first report to show that not only ILD but also emphysema has higher prevalence in patients with peripheral‐type SCLC. We also found that emphysema was more common in SCLC patients with ILD than in non‐ILD patients. Peripheral‐type SCLC patients may be at high risk of acute exacerbation of ILD or drug‐induced pneumonia, and physicians may be further aware of the possibility of these adverse events in peripheral‐type patients undergoing immunotherapy in combination with chemotherapy.

In conclusion, our study showed that SCLC could exhibit a variety of patterns on imaging, and the peripheral origin of tumors was dominant as compared to a central origin. Furthermore, peripherally located tumors were correlated with a high prevalence of emphysema and ILD.

## CONFLICT OF INTEREST

The authors declare that they have no conflict of interest.
